# Dissimilar Welding of Magnesium Alloys and Aluminum Alloys by Explosive Welding

**DOI:** 10.3390/ma18051013

**Published:** 2025-02-25

**Authors:** Mami Mihara-Narita, Konosuke Asai, Hisashi Sato, Yoshimi Watanabe, Isao Nakatsugawa, Naobumi Saito, Yasumasa Chino

**Affiliations:** 1 Department of Physical Science and Engineering, Nagoya Institute of Technology, Nagoya 466-8555, Japansato.hisashi@nitech.ac.jp (H.S.); watanabe.yoshimi@nitech.ac.jp (Y.W.); 2 National Institute of Advanced Industrial Science and Technology (AIST), Nagoya 463-8560, Japan; i.nakatsugawa@aist.go.jp (I.N.); naobumi-saito@aist.go.jp (N.S.); y-chino@aist.go.jp (Y.C.)

**Keywords:** explosive welding, dissimilar metal bonding, Mg alloy, Al alloy, interface, interlayer

## Abstract

Welding of dissimilar magnesium alloys and aluminum alloys is challenging due to the formation of interlayers composed of brittle intermetallic compounds (IMCs) at the bonding interface, which reduces the bonding strength. In our studies, we applied explosive welding to facilitate dissimilar welding of magnesium alloys and aluminum alloys. This method utilized a high-speed impact from an explosive to bond magnesium alloys and aluminum alloys in a short time, effectively suppressing the formation of the interlayer. Our research confirmed the presence of a thin interlayer of the γ-Mg_17_Al_12_ phase at the interface of the cladding plates. The alloy compositions of both magnesium alloys and aluminum alloys influenced the thickness of this interlayer. Furthermore, annealing of the cladding plates increased the thickness of the interlayer, resulting in the formation of the aluminum-rich β-Al_3_Mg_2_ phase on the aluminum alloy side after annealing at 473 K. The formation of the brittle β-Al_3_Mg_2_ phase led to crack initiation, which reduced the shear strength. In terms of corrosion resistance, the corrosion weight loss of the explosively welded cladding plates was slightly less than that of mechanically fastened samples. Therefore, it can be concluded that explosive welding is highly effective for bonding magnesium alloys to aluminum alloys.

## 1. Introduction

Recently, lightweight materials have been increasingly in demand in the automotive manufacturing industry for fuel efficiency and reduction in CO_2_ emissions. Aluminum alloys, with their low density, excellent corrosion resistance, good workability, and high thermal and electrical conductivity, are widely utilized in the automotive industry. To enhance lightweighting and fuel efficiency, some conventional steel components have been substituted with aluminum alloy parts in automobiles. Currently, multi-materialization, where various materials are utilized in appropriate locations, is attracting attention [1]. To realize this trend of multi-materialization, it is necessary to prepare suitable joining methods for various material combinations.

Magnesium alloys are the lightest practical alloys and have desirable qualities such as good castability, hot formability, and recyclability [2]. By combining magnesium and aluminum alloys in a hybrid structure, we can expand the use of these alloys to achieve even greater weight reduction, material substitution at the component level, and the joining of parts and functions [2]. Given the vast potential for dissimilarly joined magnesium/aluminum alloys in numerous applications, it is imperative to prioritize research and development of joining technology.

**Figure 1:**
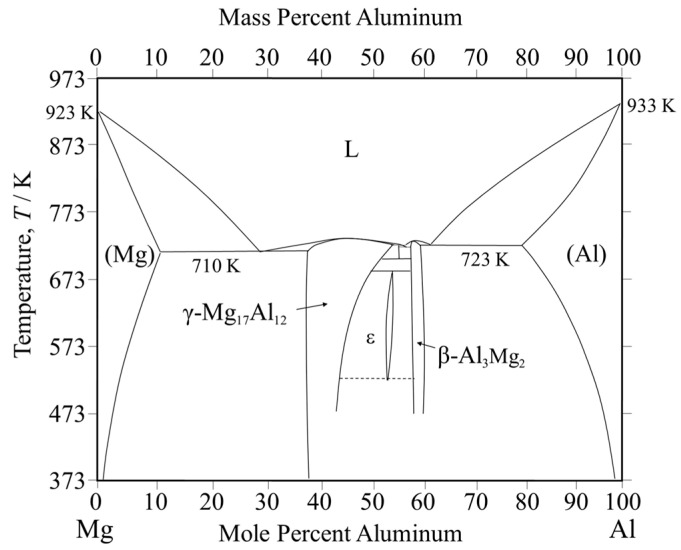
Equilibrium phase diagram of the Mg-Al system.

The welding of dissimilar magnesium and aluminum alloys has a problem due to the formation of intermetallic compounds (IMCs) [1]. Common welding techniques, including gas tungsten arc welding [3] and laser beam welding [4], often result in the formation of various types of IMCs in the weld zone, which adversely affect the quality of the welding. To overcome this issue, numerous alternative welding methods have been developed, such as solid-state welding techniques like diffusion bonding [5], ultrasonic welding [6,7], resistance spot welding [8], and friction stir welding (FSW) [1,9]. These alternative methods can help mitigate the formation of IMCs and preserve the weld’s structural integrity. FSW is a solid-state welding method, which was conducted below the melting temperature of the alloy, thereby avoiding various defects which may arise from the solidification of metals. For the welding of dissimilar alloys in the solid state such as FSW and diffusion bonding, Liu et al. [10] stated that the strength of the magnesium alloy and aluminum alloy joint increases by reducing reaction and energy, improving IMC distribution and variety. Nonetheless, the welding of dissimilar magnesium and aluminum alloys can still cause brittle intermetallic compounds at the interface, which ultimately weaken the joint [11]. The Mg-Al phase diagram (Figure 1 [12]) reveals that β-Al_3_Mg_2_ and γ-Mg_17_Al_12_ IMCs tend to form during welding.

In our studies, we have used the explosive welding technique to improve dissimilar welding of magnesium and aluminum alloys [13,14,15,16]. Explosive welding is a type of solid-state welding which uses the high-speed impact produced by explosives to bond materials in a short period [17]. Consequently, the formation of IMCs can be minimized using this method. This technique has already found practical application in the dissimilar welding of steel and aluminum alloys, with joints used as structural components for ships [17]. Although explosively welded magnesium and aluminum alloys have not been put into practical use, there has been some extensive research in recent years [18,19,20,21,22,23,24].

Yan et al. [19] conducted a study on the AZ31 (Mg-Al-Zn series magnesium alloy)/A7075 (Al-Zn-Mg series aluminum alloy) cladding plate, focusing on its microstructure and mechanical properties. Their results revealed a localized diffusion process, with a diffusion layer of approximately 3.5 μm in thickness, which facilitated the metallurgical bonding between AZ31 and A7075. In another study, Sahul et al. [20] investigated the effects of annealing on the interfacial microstructure and bonding strength of the AZ31 magnesium alloy/AW5754 (Al-Mg series aluminum alloy) cladding plate. Their findings indicated that higher annealing temperatures and longer durations increased the thickness of the intermetallic compound layer, while simultaneously reducing the joint strength. Furthermore, Zhang et al. [21] observed elongated grains with a deformed structure on the aluminum alloy side of the AZ31 magnesium alloy/A6061 (Al-Mg-Si series aluminum alloy) cladding plate. In contrast, recrystallization with equiaxed grains was observed on the magnesium alloy side. These differences were attributed to variations in crystal structure, thermal conductivity, and plastic deformability between magnesium and aluminum alloys [21].

To optimize the use of explosively welded magnesium alloy/aluminum alloy cladding plates, it is essential to comprehensively analyze their interfacial microstructure, mechanical and corrosion-resistant properties, along with the residual stress distributions at the interface. Our previous studies have investigated the properties of magnesium alloy/aluminum alloy cladding plates fabricated by explosive welding [13,14,15,16]. This article provides an overview of our findings and identifies key areas for future research aimed at advancing the development of explosively welded magnesium alloy/aluminum alloy cladding plates. Section 2 describes the explosive welding technique used in our study. In Section 3, we examine the characteristic metallographic structure at the interface of AZ31 magnesium alloy and A6005C (Al-Mg-Si series) aluminum alloy cladding plates, highlighting the formation of a thin interlayer during the explosive welding process. Section 4 discusses the influence of the composition of the magnesium alloy on the formation of the interlayer and the mechanical properties of the cladding plates. A comparison of the corrosion resistance between explosively welded and mechanically fastened plates is also presented. Section 5 briefly addresses the influence of aluminum alloy composition on interlayer formation and the mechanical properties of the cladding plates. In Section 6, we evaluate the effects of post-weld annealing on the formation of the interlayer and the mechanical properties of the cladding plates. Finally, Section 7 provides our concluding remarks.

## 2. Method of Explosive Welding

**Figure 2:**
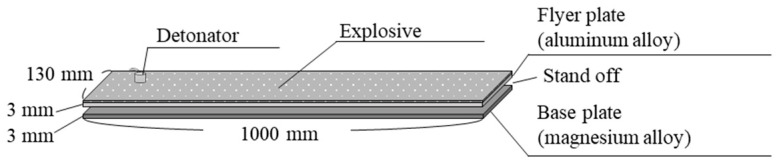
Experimental setup for the explosive welding process.

In our studies, magnesium alloys and aluminum alloys were used as base and flyer plates, respectively [13,14,15,16]. The samples were cleaned using solvents such as ethanol to remove grease and contaminants. After cleaning, mechanical polishing was performed. The surface of the sample intended for explosive welding should have a roughness (Ra) of approximately a few micrometers. During explosive welding, the base and flyer plates were placed with a certain gap, and an explosive with a constant thickness was laid over the entire area of the flyer plate, as shown in Figure 2. By detonating the explosive (Anmonium Nitrate Fuel Oil explosive (ANFO) type) with a detonator, the flyer plate collided with the base material, completing the joining of the two alloys. Both the cladding and the base material show high deformation velocity and fluid-like behavior during explosive welding, which generates a metal jet at the collision point [25]. As a result, the oxide film on the metal surface is removed, and the clean surfaces are instantly joined together [26].

## 3. Characterization of Metallographic Structure at the Interface

In this section, the microstructure of the explosively welded AZ31 magnesium alloy/A6005C aluminum alloy cladding plate is described. AZ31 consists mainly of magnesium (approximately 90%) with aluminum, zinc, and small amounts of other elements such as manganese and silicon. It is the most used magnesium alloy in wrought materials. It is also relatively easy to process, often used in casting, rolling, and extrusion processes. A6005C is part of the 6000 series aluminum alloy, which primarily consists of aluminum, silicon, and magnesium. This alloy is known for its excellent balance of strength, corrosion resistance, and good workability, widely used due to its versatile properties, and is particularly popular for structural applications. In this section, the results of applying explosive welding to the joining of AZ31 and A6005C, which possess the characteristics mentioned above, are presented. The samples were prepared from extruded materials of the AZ31 magnesium alloy and the A6005C aluminum alloy, with a thickness of 3 mm, a width of 130 mm, and a length of 1000 mm. The chemical compositions of extruded materials are shown in Table 1.

### 3.1. Interfacial Microstructure

**Figure 3:**
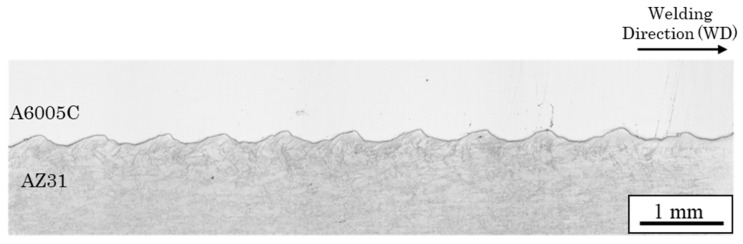
Optical microscope image at the interface of the explosively welded AZ31/A6005C cladding plate [13].

**Figure 4:**
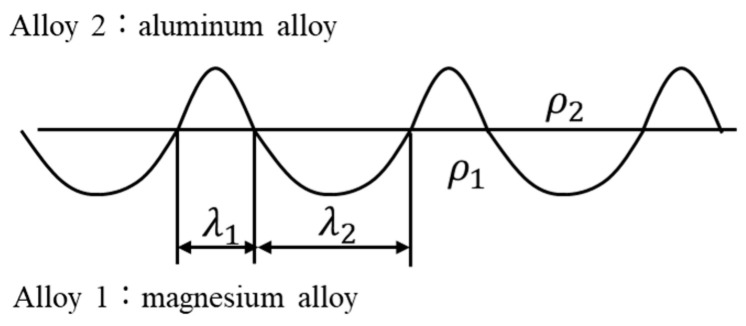
Schematic illustration showing the half-wavelengths of the wavy interfaces (*λ*_1_ and *λ*_2_) and the densities (*ρ*_1_ and *ρ*_2_), respectively, at the interface of dissimilar materials 1 and 2 in joined material.

Figure 3 shows a cross-sectional optical image of the joint interface. Typically, explosively welded materials have a wavy interface [19], which was also observed in this sample. Compared to a smooth interface, the wavy interface enhances the shear strength of the joints by increasing the area of interfacial contact. The waveforms on the magnesium alloy and aluminum alloy sides differ, unlike a sinusoidal wave with a constant wavelength. Reid et al. suggested that differences in material properties affect the shape of the interface [27]. In materials 1 and 2, with different properties, the half-wavelengths are *λ*_1_ and *λ*_2_, and the densities are *ρ*_1_ and *ρ*_2_, respectively (Figure 4). The relationship between wavelength and density is expressed as follows [27].

The densities of the AZ31 magnesium alloy and A6005C aluminum alloy are 1.78 Mg/m^3^ and 2.70 Mg/m^3^, respectively. In Figure 4, material 1 and material 2 refer to AZ31 and A6005C, and *λ*_1_ and *λ_2_* correspond to *λ*_AZ31_ and *λ*_A6005C_, respectively. From Equation (1), the relationship *λ*_A6005C_ = 0.81*λ*_AZ31_ is obtained. The experimental results show that the relationship is *λ*_A6005C_ = 0.87*λ*_AZ31_, which is closely aligned with the theoretical relationship.

(1) $$\frac{\lambda_{2}}{\lambda_{1}}=\sqrt{\frac{\rho_{2}}{\rho_{1}}}$$

**Figure 5:**
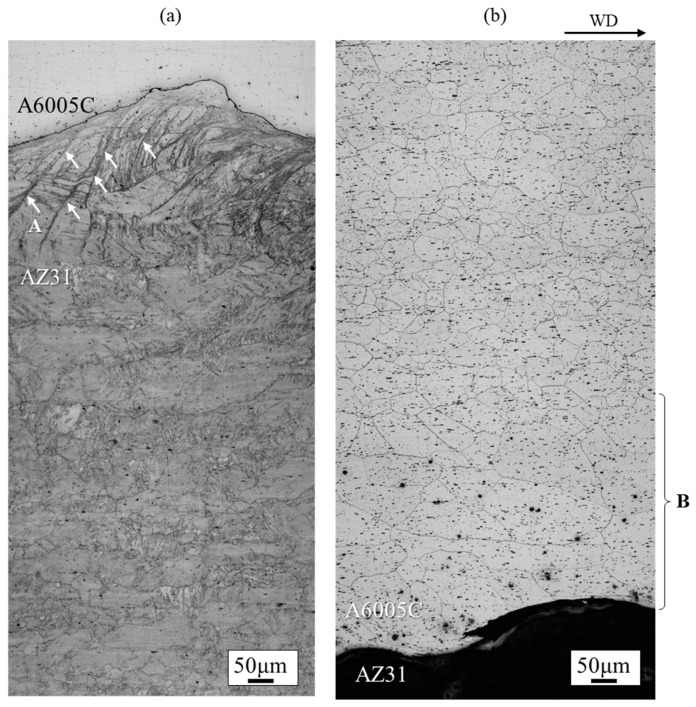
Optical microscope images at the (**a**) AZ31 side and (**b**) A6005C side interface of the explosively welded AZ31/A6005C cladding plate [13]. In (**a**), A indicates the shear bands observed on the AZ31 side, while in (**b**), B indicates the region where the plastic flow of the grains was observed on the A6005C side.

A wide-area microstructural observation of the cross-section parallel to the welding direction of the AZ31/A6005C cladding plate is shown in Figure 5. On the AZ31 side shown in Figure 5a, shear bands (indicated by the arrows at location A in Figure 5a) were observed up to 250 μm below the bonding interface, and deformation twins were observed throughout the cross-section, which were not seen in the extruded material. This suggests that deformation twins formed during explosive welding, probably as a result of high-strain deformation, where adiabatic shear bands form first, followed by twin deformation to accommodate unmanageable strains. On the A6005C side shown in Figure 5b, the plastic flow of the grains (in the region marked B in Figure 5b) was observed near the joint interface due to explosive welding.

During explosive welding, an adiabatic temperature gradient is assumed to occur locally at the interface, leading to intense deformation in the high temperature region [28]. The average strain rate during explosion is between 10^4^ s^−1^ to 10^10^ s^−1^ [25]. The rise in temperature during explosive welding can exceed the melting point of AZ31 (approximately 823 K) [13], which is attributed to the high deformation resistance at the grain boundaries and the presence of precipitates and impurities. Various theories for the wavy interface in explosively welded cladding plates have been proposed [29]. In our study, shear bands were found at the bonding interface, and deformation twinning was observed throughout the thickness, suggesting that the wavy shape results from shear flow along the welding direction and displacement restraint in the vertical direction [30,31].

### 3.2. Characteristics of the Interlayer at the Interface

**Figure 6:**
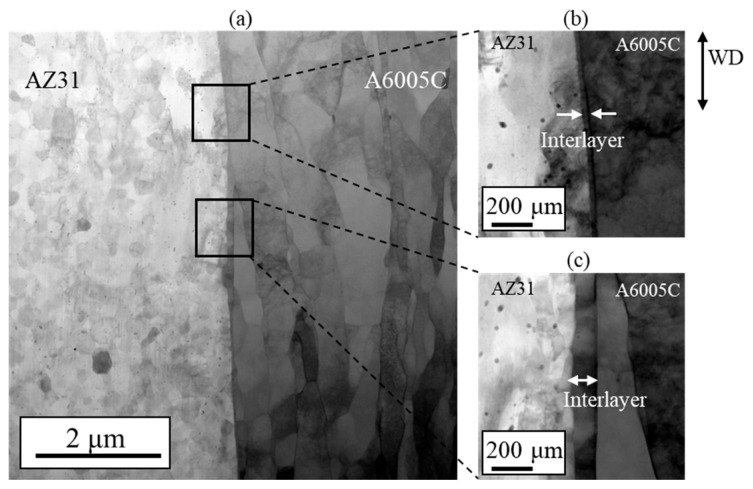
STEM images at the interface of the explosively welded AZ31/A6005C cladding plate [13].

In the explosive welding process, both deformation and cooling rates are typically high, which has led to reports suggesting that IMCs are not present at the bonding interface, even when dissimilar materials are welded. Based on the observations shown in Figure 3, the presence of IMCs at the bonding interface could not be conclusively identified. However, a detailed morphological examination using a scanning transmission electron microscope (STEM) revealed a thin interlayer between A6005C and AZ31, as shown in Figure 6a. In particular, no oxide layers were detected at the bonding interface, and metal jets were emitted from metal surfaces, effectively removing surface oxides and facilitating bonding on freshly exposed surfaces. The thickness of the interlayer was approximately 30 nm to 40 nm in the region shown in Figure 6b and approximately 80 nm to 95 nm in the area depicted in Figure 6c, with a non-uniform distribution. Additionally, it was observed that the interface protruded towards the magnesium side.

**Figure 7:**
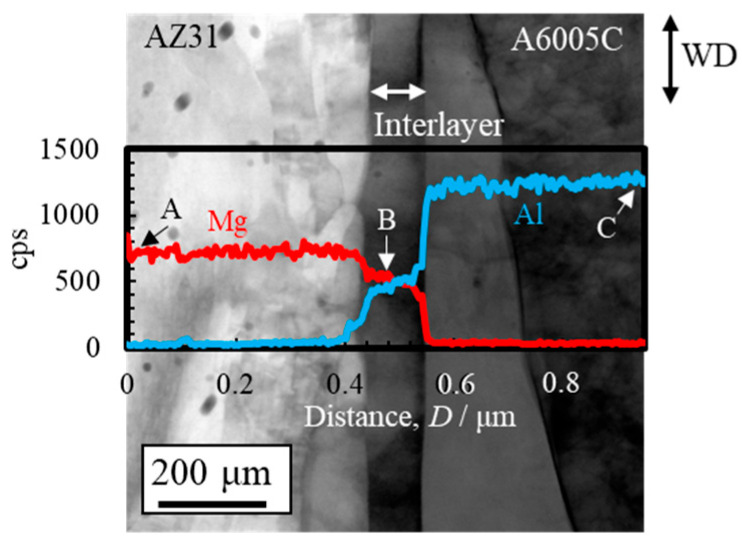
STEM image and line profiles of magnesium and aluminum compositions near the bonding interface of the explosively welded AZ31/A6005C cladding plate [13]. (Figure is modified from the original version).

A STEM image, along with line profiles of magnesium and aluminum compositions near the bonding interface, is shown in Figure 7. The region between point A (AZ31) and the interface shows a high concentration of magnesium, while the region between point C (A6005C) and the interface is rich in aluminum. Near point B, alternating concentrations of magnesium and aluminum are observed. Additionally, the gradient in the concentrations of magnesium and aluminum corresponds to the thickness of the interlayer. This gradient region is presumed to represent the interdiffusion zone. The diffusion coefficients for the interdiffusion of aluminum to magnesium and from magnesium to aluminum at 673 K are 5.5 × 10⁻^16^ m^2^/s and 9.4 × 10⁻^15^ m^2^/s, respectively [32]. Based on these values, it can be inferred that a greater number of atoms are transferred from magnesium to aluminum during explosive welding. As a result, it is assumed that the bonding interface shifts toward the magnesium side as a result of the diffusion of atoms from magnesium into aluminum.

**Figure 8:**
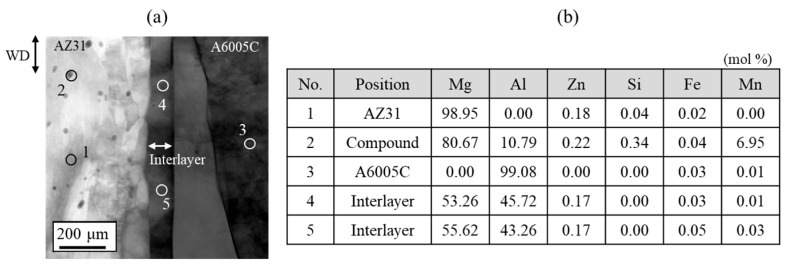
(**a**) Position of the chemical composition analysis (1: AZ31, 2: compound in AZ31, 3: A6005C, 4 and 5: interlayer) and (**b**) results of the chemical composition analysis for the explosively welded AZ31/A6005C cladding plate [13].

Figure 8 shows the results of the chemical composition analysis performed near the bonding interface. The measurement positions and corresponding results are shown in Figure 8a and Figure 8b, respectively. In the interlayer, the magnesium content slightly exceeds that of aluminum. This suggests that aluminum from AZ31 and magnesium from A6005C are concentrated in the interlayer at the bonding interface. Based on the equilibrium phase diagram of the Mg–Al system shown in Figure 1, the presence of β-Al_3_Mg_2_ and γ-Mg_17_Al_12_ phases is expected in regions with intermediate levels of magnesium and aluminum. The diffusion distances can be calculated using Equation (2), where *x* represents the diffusion distance, *D* is the diffusion coefficient, and *t* is the time. The diffusion coefficients of β-Al_3_Mg_2_ and γ-Mg_17_Al_12_ are 2.0 × 10⁻^16^ m^2^/s and 2.9 × 10⁻^15^ m^2^/s, respectively [32].

(2) $$x=\sqrt{Dt}$$

In the explosive welding process, the cooling rate of the sample is rapid. For example, considering the formation of the diffusion layer within 0.1 s, the diffusion lengths of the β-Al_3_Mg_2_ and γ-Mg_17_Al_12_ phases are 17 nm and 44 nm, respectively. Therefore, there is a possibility of the formation of both phases during explosive welding. It is also predicted that the γ-Mg_17_Al_12_ phase is likely to form prior to the β-Al_3_Mg_2_ phase. Future work will focus on elucidating the formation and growth mechanisms of these phases, particularly in relation to the dynamic recrystallization behavior during the cooling process in explosive welding, as well as estimating the activation energy. Furthermore, the composition of the interlayer at the bonding interface needs to be evaluated. A proposed model suggests instantaneous melting and solidification, independent of heat conduction, during collision of two metal plates during the joining process [33]. This can be expressed by the following equation.

(3) $$\frac{M_{AZ31}}{M_{A6005C}}=\frac{c_{A6005C}\left(T_{mA6005C}-T_{0}\right)+H_{A6005C}}{c_{1}\left(T_{mAZ31}-T_{0}\right)+H_{AZ31}}$$

where *M* is the reaction composition, *c* is the specific heat, *T_m_* is the melting point, *T*_0_ is the room temperature, and *H* is the latent heat. The specific heats of magnesium and aluminum are 1.045 g^−1^K^−1^ and 0.899 Jg^−1^K^−1^, respectively [34]. The melting points of magnesium and aluminum are assumed to be 923 K and 943 K, respectively. *T*_0_ is 293 K, and the latent heats of magnesium and aluminum at 973 K are 230 Jg^−1^ and 394 Jg^−1^, respectively [34]. Substituting these constants into Equation (3), we obtain the relationship *M*_A6005C_ = 1.2*M*_AZ31_. At analysis point No. 4 in Figure 8b, the measured magnesium and aluminum concentrations at the interface nearly satisfy this relationship. Therefore, it is considered that the formation of the interlayer results from instantaneous melting and solidification during explosive welding, independently of heat conduction.

**Figure 9:**
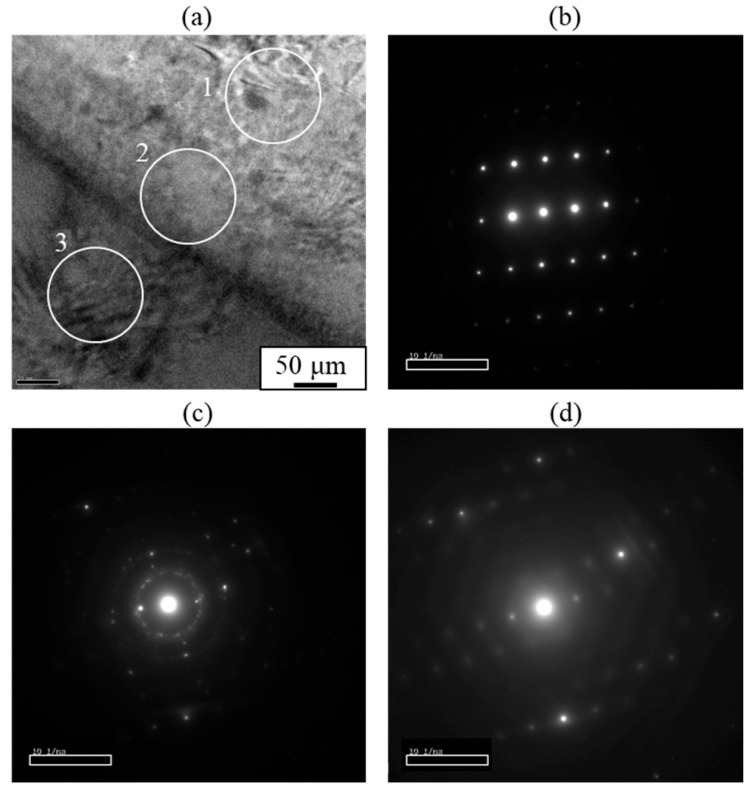
(**a**) Analysis areas and (**b**–**d**) selected area diffraction patterns obtained at the interface of the explosively welded AZ31/A6005C cladding plate. Each selected area diffraction pattern corresponds to (**b**) A6005C side (area 1), (**c**) interlayer (area 2), and (**d**) AZ31 side (area 3), respectively [13].

Based on these observations, it is suggested that the γ-Mg_17_Al_12_ phase exists within the interlayer at the bonding interface of the explosively welded AZ31/A6005C cladding plate. Conversely, as shown in Figure 8, a concentration gradient is observed within the interlayer, indicating the presence of a diffusion layer. Additionally, from the selected area’s diffraction pattern near the interface, shown in Figure 9, a halo pattern, in addition to lattice diffraction spots, was observed inside the interlayer, suggesting the coexistence of crystalline and amorphous structures. Therefore, it is inferred that IMCs and amorphous structures coexist to form the interlayer. The diffraction patterns shown in Figure 9c closely resemble those of both β-Al_3_Mg_2_ and γ-Mg_17_Al_12_ phases, making it impossible to conclusively identify the phases from the diffraction data alone.

### 3.3. Grain Refinement in Magnesium Alloy Side

**Figure 10:**
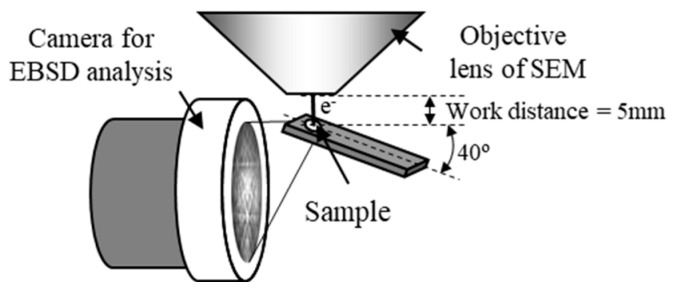
Layout of the sample and measuring device in the transmission EBSD method [13,35].

The crystal orientation distribution near the interface can be investigated by electron backscatter diffraction (EBSD). The grain size near the interface on the AZ31 side ranged from 50 nm to 1 μm. However, it is difficult for the conventional EBSD method to measure the orientation of such fine grains. Therefore, we performed high-resolution crystal orientation analysis in micro-regions using the transmission EBSD (t-EBSD) method [35]. In this approach, crystal orientation analysis can be conducted on a thin film sample by EBSD, as illustrated in Figure 10. This method offers the advantage of higher spatial resolution compared to conventional EBSD techniques, enabling rapid measurement of crystal orientation distribution for nano-scale structures in thin film samples.

**Figure 11:**
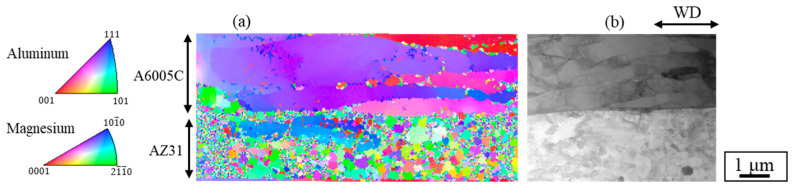
IPF map of t-EBSD near the interface of the explosively welded AZ31/A6005C cladding plate [13].

Figure 11 shows the inverse pole figure (IPF) map near the bonding interface, obtained using the t-EBSD method (Figure 11a) with the STEM image (Figure 11b). Coarse grains were observed on the A6005C side, while grain refinement was evident near the interface on the AZ31 side. The grain size measured in regions with a high confidence index (CI > 0.05) on the AZ31 side ranged from 50 nm to 1 μm. The initial grain size of AZ31 was approximately 50 μm; therefore, it can be inferred that the grains on the AZ31 side of the bonding interface underwent refinement as a result of explosive welding. On the A6005C side, low-angle grain boundaries were observed within the coarse grains. The stacking fault energies of magnesium and aluminum are 78 mJ/m^2^ and 200 mJ/m^2^, respectively [36]. Consequently, on the A6005C side, which has a relatively high stacking fault energy, coarse grains are predominantly observed due to recovery mechanisms. This suggests that the grain structure observed near the bonding interface was likely influenced by recovery and recrystallization processes induced by high-speed deformation during explosive welding.

In bulk materials, recrystallization is typically enhanced in highly deformed materials. The recrystallization behavior of magnesium alloys can be elucidated by considering the Zener–Hollomon parameter (Z-parameter) [37].

(4) $$Zd^{m}=A$$

Here, *d* is the grain size, *m* is the particle size index, A is a constant, and the *Z* parameter is expressed by the following equation.

(5) $$Z=\dot{\varepsilon }exp\left(\frac{Q}{RT}\right)$$

where $\dot{\varepsilon }$ is strain rate, *Q* is activation energy, *T* is temperature, and *R* is gas constant. The relationship between the crystal grain size (*d_rec_*) and the initial grain size (*d*_0_) in recrystallization of magnesium alloys is expressed by Equation (6) based on Equation (5) and the experimental results.

(6) $$\left(\frac{d_{rec}}{d_{0}}\right)=10^{3}\times Z^{-\frac{1}{3}}$$

Here, the strain rate is assumed to be 1.6 × 10^6^/s in the evaluation of temperature rise due to slip deformation near the interface [25]. Furthermore, when *Q* is 165 kJ/mol, which is the activation energy of AZ31, *T* is 673 K, and R is 8.31 J/mol K, the *Z* parameter is calculated from Equation (5) to be 1.89 × 10^17^ s^−1^ [16]. Substituting this into Equation (6), *d_rec_*/*d*_0_ becomes 1.9 × 10^−3^. Assuming that the initial grain size *d_0_* is approximately 50 μm [17], *d_rec_* is calculated to be 20 nm to 30 nm, which is consistent with the fine grain size observed on the AZ31 side in Figure 11. A slip line is also observed at the bonding interface, and recrystallization due to adiabatic shear deformation occurs on the AZ31 side (Figure 5). From these facts, fine recrystallized grains (subgrains) are considered to have been generated on the AZ31 side by explosive welding. The temperature was assumed to be 673 K and the strain rate was assumed to be 1 × 10^6^ s^−1^ for the analysis; however, the details of the absolute value of the grain size change need further investigation.

## 4. Effect of Magnesium Alloy Composition

In the previous section, interfacial microstructures and properties of the explosively welded cladding plates were characterized in the specific alloys. AZ31 is the most used magnesium alloy in wrought materials. AZ61 and AZ80 with increased aluminum additions have better strength properties than this, and in industrial applications different types of alloys are selected for different applications. However, it is not well understood how different compositions of the magnesium alloy change the properties of the explosively welded magnesium alloy/aluminum alloy cladding plates. Therefore, in this section, the effects of the compositions of magnesium alloy on the interfacial microstructure, corrosion resistance, and mechanical properties of explosively welded cladding plates are investigated [14]. Samples for this study were prepared from extruded materials of AZ31, AZ61, and AZ80 magnesium alloys, and A6005C aluminum alloys, with a thickness of 3 mm, a width of 130 mm, and a length of 1000 mm. The chemical compositions of extruded materials are shown in Table 2.

### 4.1. Interfacial Microstructure

**Figure 12:**
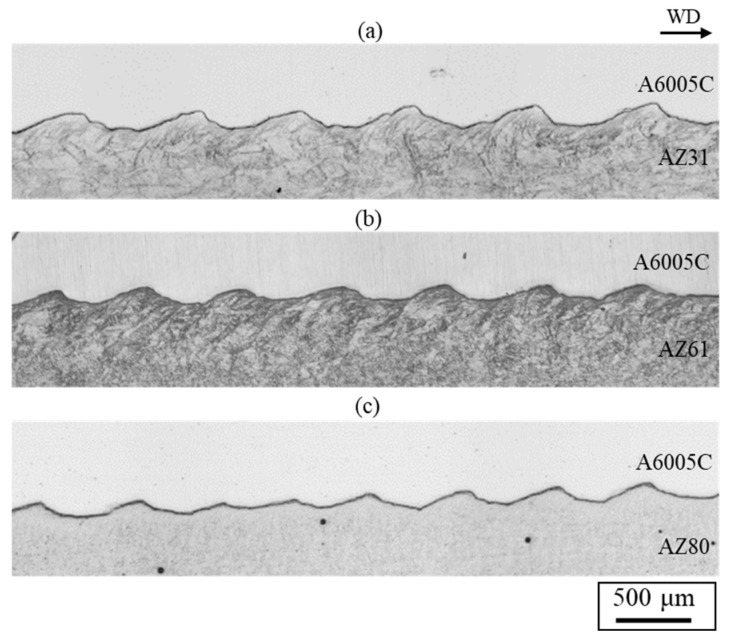
Optical microscope images for explosively welded (**a**) AZ31/A6005C, (**b**) AZ61/A6005C, and (**c**) AZ80/A6005C cladding plates [14].

Figure 12 shows cross-sectional optical images for explosively welded (a) AZ31/A6005C, (b) AZ61/A6005C, and (c) AZ80/A6005C cladding plates, respectively. The interface in all samples exhibited a wavy shape. The densities of the AZ61 and AZ80 magnesium alloys are 1.80 Mg/m^3^ and 1.81 Mg/m^3^, respectively [34]. From Equation (1), the relationship of *λ*_A6005C_ = 0.82*λ*_AZ61_ and *λ*_A6005C_ = 0.82*λ*_AZ81_ is obtained. The experimental results show the relationship between *λ*_A6005C_ = 0.77*λ*_AZ61_ and *λ*_A6005C_ = 0.82*λ*_AZ81_. No significant differences in values were observed between samples. In explosively welded materials, there is a difference in wave height between the detonation area and the ends of the detonation area, and the wave height is generally larger at the ends of the detonation area. In this study, the wave shape was measured by taking a sample from the middle part of the explosively welded materials, and the variation of the wave shape depending on the measurement position was not considered.

**Figure 13:**
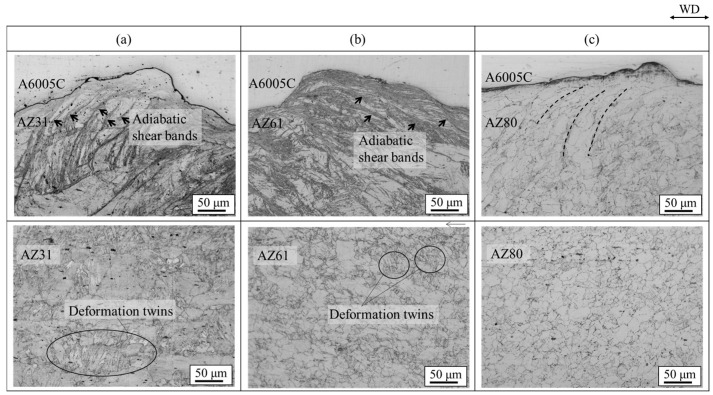
Enlarged optical microscope images of explosively welded (**a**) AZ31/A6005C, (**b**) AZ61/A6005C, and (**c**) AZ80/A6005C cladding plates [15]. Arrows indicate the adiabatic shear bands observed on the magnesium alloy side. Dashed lines indicate the region where the plastic flow of the grains was observed on the aluminum alloy side. (The figure is modified from the original version).

Enlarged optical microscope images for explosively welded (a) AZ31/A6005C, (b) AZ61/A6005C, and (c) AZ80/A6005C cladding plates are shown in Figure 13. They show the microstructure of the magnesium alloy side for each sample. In the explosively welded AZ31/A6005C and AZ60/A6005C cladding plates, the adiabatic shear bands are prominently visible at the interface on the magnesium alloy side, as detailed in Section 3. In these cladding plates, deformation twins were observed across the entire cross-section parallel to the welding direction. On the contrary, in the explosively welded AZ80/A6005C cladding plate, deformation twins were only observed in the region up to approximately 500 μm below the bonding interface.

**Figure 14:**
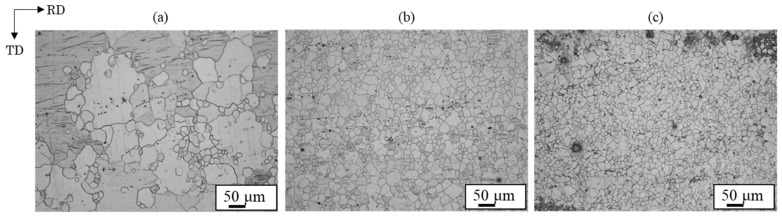
Optical microscope images for extruded (**a**) AZ31, (**b**) AZ61 and (**c**) AZ80 samples [14].

Microstructures of extruded magnesium alloy sheets of AZ31, AZ61, and AZ80 are presented in Figure 14. The grain size decreased as the aluminum concentration in the magnesium alloy increased. AZ31 had a mixed grain structure of coarse and fine grains of 10~100 µm, while AZ61 and AZ80 had a uniform structure of about 20 µm. The grain boundaries play a crucial role in the deformation twinning, as they serve as nucleation sites for the deformation twinning [26,38]. Stress induced by deformation twinning has been reported to follow the Hall–Petch relation [39,40]. Therefore, grain refinement to a few microns reduces the density of deformation twins. Formation of deformation twins was suppressed in AZ80 compared to AZ31 and AZ61 due to its finer grains. Additionally, the amount of plastic strain generated during bonding increases near the bonding interface, with the strain distribution decreasing with distance from the interface [21]. These factors contributed to the observation of deformation twins up to a certain distance from the bond interface.

### 4.2. Characteristics of the Interlayer at the Interface

**Figure 15:**
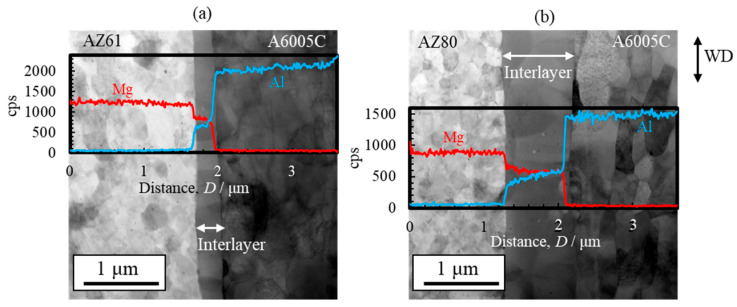
STEM images and line profiles of magnesium and aluminum compositions near the bonding interface of explosively welded (**a**) AZ61/A6005C and (**b**) AZ80/A6005C cladding plates [14]. (Figure is modified from the original version).

Figure 15 displays STEM images of the AZ61/A6005C and AZ80/A6005C cladding plates. Unlike the AZ31/A6005C cladding plate (Figure 6), where a thin, nonuniform interlayer was present at the bonding interface, the AZ61/A6005C and AZ80/A6005C cladding plates exhibit a uniform thickness of the interlayer at the interface. Specifically, the thickness of the interlayers are approximately 0.5 μm for the AZ61/A6005C (Figure 15a) and 0.7 μm for the AZ80/A6005C (Figure 15b). This indicates that the thickness of the interlayer increases as the aluminum concentration in the magnesium alloy increases. However, the thickness of the interlayer remains less than 1 μm, which is considerably smaller compared to other joining methods. This suggests that the explosive welding process is effective in minimizing the formation of the interlayer at the bonding interface.

Line profiles of magnesium and aluminum compositions near the bonding interface revealed that the gradient in magnesium and aluminum content corresponds well to the thickness of the interlayer, similar to those of the AZ31/A6005C cladding plate (Figure 7). The chemical composition analysis also confirms the formation of the γ-Mg_17_Al_12_ phase in the interlayer of both the AZ61/A6005C and AZ80/A6005C cladding plates.

### 4.3. Shear Strength

**Figure 16:**
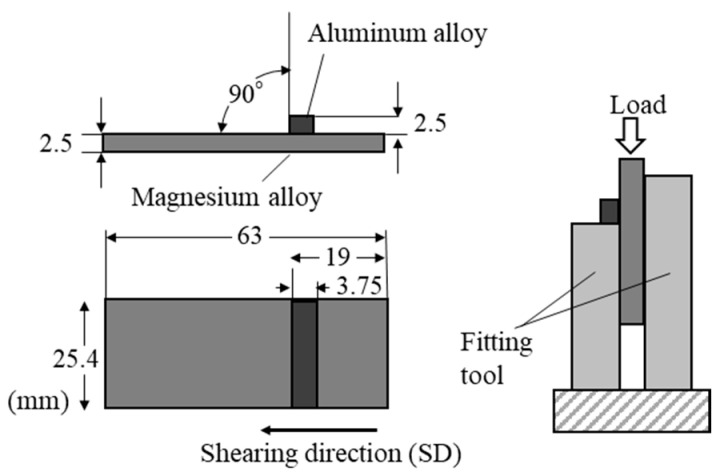
Schematic illustration for the shearing test.

To evaluate the mechanical properties of the explosively cladded plates, shear tests were conducted. Schematic illustration of the shear test setup is shown in Figure 16. The explosively cladded plates were cut to 25.4 mm wide and 63 mm long, then processed into the shape of shear test specimens (Figure 16). The magnesium alloy and aluminum alloy surfaces were machined by 0.5 mm each to produce parallelism on both sides. The flyer plate (made of aluminum alloy) was separated from the base plate (made of magnesium alloy) parallel to the joint surface by applying a load from above to the sample sandwiched between the fixtures. The crosshead speed was set to 1 mm/min, and each sample was subjected to three tests to determine the average shear strength. According to JISG0601 standards [41], the shear strength was calculated using the following formula.

(7) $$\sigma_{s}=\frac{P}{S}$$

Here, $\sigma_{s}$ is shear strength (Pa), P is load (N), and S is joint area (m^2^).

**Figure 17:**
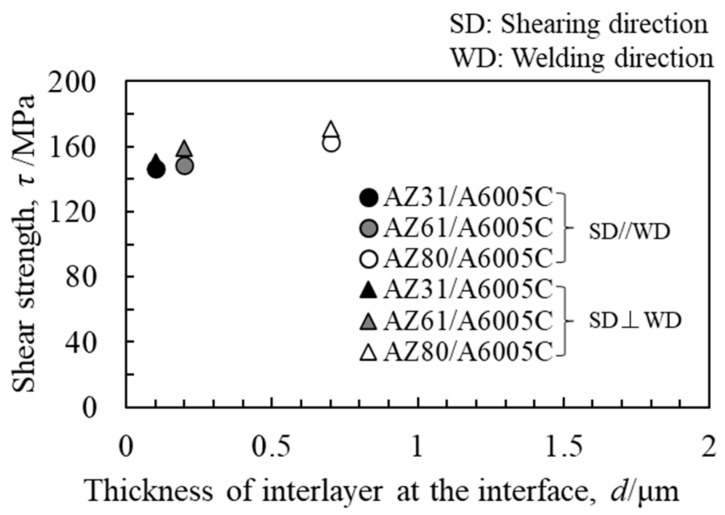
Relationship between shear strength and thickness of the interlayer for explosively welded cladding plates [14].

**Figure 18:**
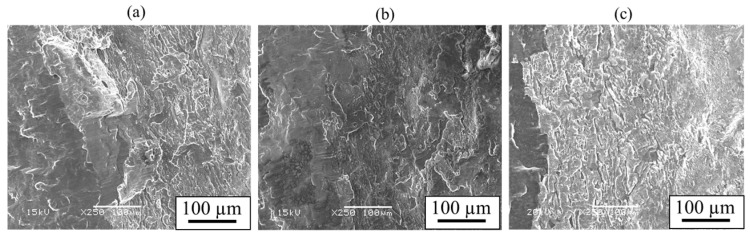
Fracture surface after shear test for the explosively welded (**a**) AZ31/A6005C, (**b**) AZ61/A6005C, and (**c**) AZ80/A6005C cladding plates [14]. (The figure is modified from the original version).

The obtained shear strength, along with the thickness of the interlayer, is presented in Figure 17. The shear strength increased on the order of AZ31/A6005C, AZ61/A6005C, and AZ80/A6005C. It was observed that the AZ80/A6005C cladding plate, where a uniformly formed interlayer was present at the interface, exhibited higher shear strength compared to the AZ31/A6005C cladding plate, where a thin and non-uniformly distributed interlayer was present at the interface. The shear strength tended to be higher when the shear direction (SD) was parallel to the welding direction (WD) than when it was perpendicular to it. Figure 18 displays the fracture surface of the specimens on the magnesium alloy side of (a) AZ31/A6005C, (b) AZ61/A6005C, and (c) AZ80/A6005C cladding plates after the shear test. In all samples, the cross-section exhibited a cleavage fracture, indicative of a brittle fracture. The river pattern on the fracture surface did not exhibit a fixed direction, indicating unclear directions for crack propagation. Traces of the interlayer were observed on the fracture surface, suggesting that cracks may have originated from there during the shear test. As discussed in Section 4.1, there were no significant differences in the waveforms at the interface between the samples, so the differences in the waveforms were not considered to affect the shear strength in this test.

### 4.4. Corrosion Resistance

**Figure 19:**
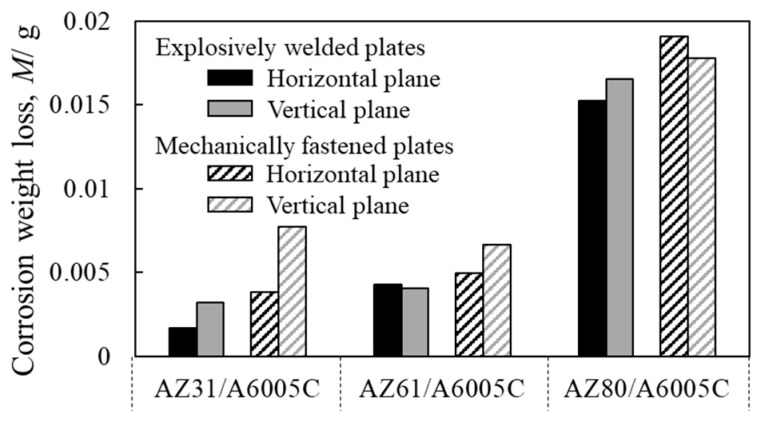
Weight loss after corrosion test for explosively welded plates and mechanically fastened plates [14]. (The figure is modified from the original version).

The corrosion resistance of explosively welded cladding plates was investigated in a saltwater immersion environment, with mechanically fastened samples included for comparison [14]. The test condition was selected based on our past study [42]. Figure 19 shows the weight loss of corrosion obtained after immersion for 4 h in explosively welded cladding plates and mechanically fastened samples. Before the corrosion test, the sample was heated at 353 K for 1 h and embedded in resin. For the corrosion test solution, a solution prepared by adding Mg(OH)_2_ to a 1 mass% NaCl solution and adjusting the pH to 9–10 was used. The test was carried out twice, and the corrosion weight loss represents the average value. The corrosion weight loss of the explosively welded cladding plates was slightly smaller than that of the mechanically fastened samples. Comparison of corrosion weight loss revealed that corrosion progressed more readily in mechanically fastened samples compared to explosively welded cladding plates. Furthermore, AZ80/A6005C, which has a higher aluminum content in the magnesium alloy, exhibited approximately twice the corrosion weight loss compared to AZ31/A6005C and AZ61/A6005C. These corrosion behavior tendencies were consistent across different test surfaces and joining methods. On the aluminum alloy side, uniform corrosion was observed in all samples.

**Figure 20:**
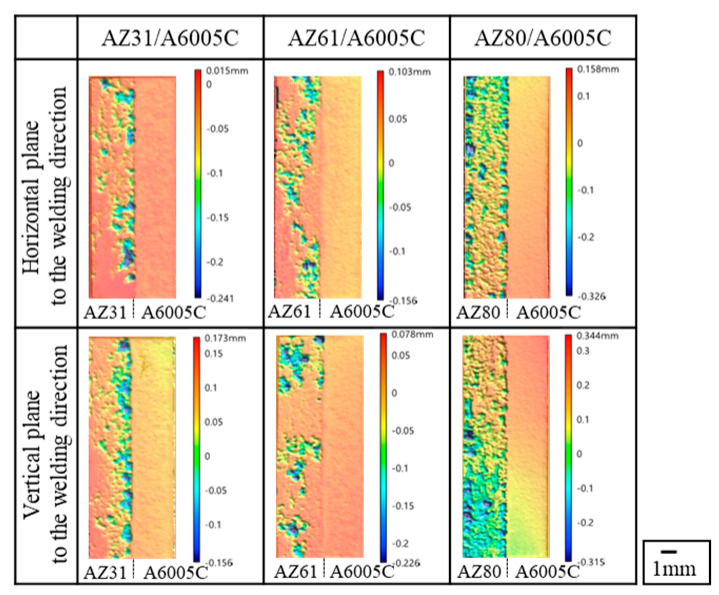
Surface profile of immersion depth after corrosion test for explosively welded cladding plates [14].

The surface profiles of the depth of corrosion in the explosively welded cladding plates were measured by a 3D optical profiler (Keyence VR-5100). The results are presented in Figure 20. The red portion of the surface profile indicates a shallower depth of corrosion, while the blue color represents a deeper depth of corrosion. In the case of AZ31/A6005C, corrosion occurred near the joint interface. For AZ61/A6005C, some areas showed corrosion in the central part, with no clear trend observed. Both samples exhibited regions where no corrosion occurred on the magnesium alloy side. In the case of the AZ80/A6005C, the entire surface magnesium of the alloy was corroded. As the aluminum concentration increases in the magnesium alloy, the corrosion potential of the alloy decreases. Consequently, the potential difference between the aluminum alloy and the magnesium alloy increases, accelerating galvanic corrosion, which is believed to account for the above-mentioned results. The uniform corrosion on the aluminum alloy side in all samples is attributed to active corrosion induced by the alkaline solution generated by the cathode reaction [43].

## 5. Effect of Aluminum Alloy Compositions

In this section, the effects of aluminum alloy compositions on the interfacial microstructure and mechanical properties of explosively welded cladding plates are investigated. Here, AZ31 and AZ80, which were also used in the previous section, were used, and the A5052 alloy (Al-Mg series aluminum alloy) with a composition that has a higher magnesium content than A6005C was newly applied for this study. A5052 is an aluminum alloy that is primarily composed of aluminum with magnesium as the main alloying element. Although it has less strength than A6005C, A5052 is widely used in structural applications where good corrosion resistance, formability, and moderate strength are required. Since different aluminum alloys are used for different applications, studies on joining materials with varying aluminum alloy species are useful. The sample size before explosive deposition is the same as in the previous section. The chemical compositions of extruded materials are shown in Table 3.

### 5.1. Interfacial Microstructure and Characteristics of the Interlayer at the Interface

**Figure 21:**
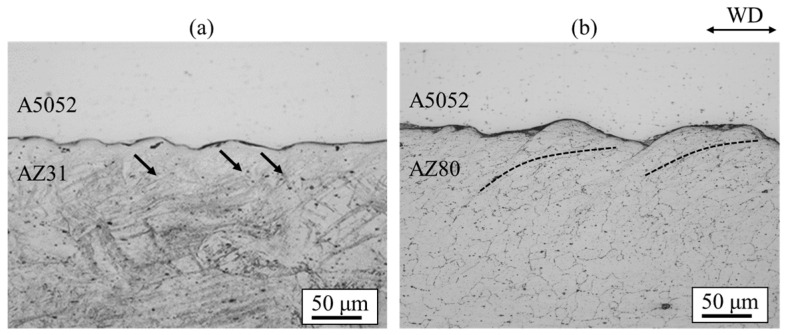
Optical microscopy images for explosively welded (**a**) AZ31/A5052 and (**b**) AZ80/A5052 cladding plates [15]. Arrows indicate the adiabatic shear bands observed on the magnesium alloy side for AZ31/A5052 cladding plate. Dashed lines indicate the region where the plastic flow of the refined grains was observed on the magnesium alloy side for AZ80/A5052 cladding plate.

Figure 21 shows the cross-sectional optical images of AZ31/A5052 and AZ80/A5052 cladding plates. The interface of all samples exhibited a wavy shape. The densities of the A5052 aluminum alloy is 2.68 Mg/m^3^. From Equation (1), the relationship of λ_A5052_ = 0.81λ_AZ31_ and λ_A5052_ = 0.82λ_AZ80_ is obtained. The experimental results show the relationship of λ_A5052_ = 0.83λ_AZ31_ and λ_A5052_ = 0.77λ_AZ80_, which aligns closely with the theoretical relationship. Compared to the wavy interface observed in the cladding plate utilizing the A6005C aluminum alloy (Figure 12), the wavelength and amplitude of the waves were apparently small. It has been reported that the shape of the wavy interface varies depending on the impact angle of the base plate and the flyer plate. In this study, the conditions of explosive welding were consistent in all experiments; however, it is presumed that the difference in the bending moment caused by the mechanical properties of the flyer plate alters the impact angle [25]. From the results of tensile tests, the work hardening coefficient n can be obtained using the following equation.

(8) $$n=ln(\varepsilon_{n}+1)$$

where $\varepsilon_{n}$ represents the strain value at maximum stress. The extruded material showed that the *n*-values of A6005C and A5052 were 0.09 and 0.12, respectively. Since A5052 has a higher *n*-value than A6005C, it is easier for A5052 to work harden than it is for A6005C when the same stress is applied. Therefore, A5052 is less likely to deform than A6005C due to work hardening during collision, resulting in smaller wave sizes. In the AZ31/A5052 cladding plate, adiabatic shear bands are observed at the interface on the magnesium alloy side, as indicated by the arrows in Figure 21. On the contrary, in the AZ80/A5052 cladding plate, the grains were refined near the bonding interface on the AZ80 side. This refinement is believed to be caused by recrystallization due to adiabatic shear deformation during explosive welding.

**Figure 22:**
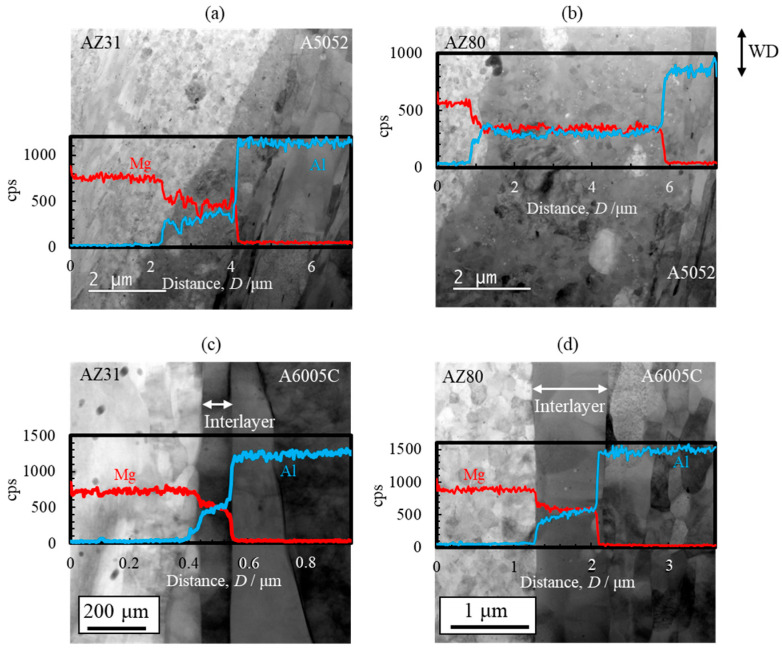
STEM images and line profiles of magnesium and aluminum compositions near the bonding interface of explosively welded (**a**) AZ31/A5052, (**b**) AZ80/A5052, (**c**) AZ31/A6005C, and (**d**) AZ80/A6005C cladding plates [15]. (The figure is modified from the original version).

STEM images and line profiles of magnesium and aluminum compositions near the bonding interface of the AZ31/A5052 and AZ80/A5052 cladding plates are shown in Figure 22a and Figure 22b, respectively. For comparison, the results of AZ31/A6005C and AZ80/A6005C cladding plates are also shown in Figure 22c and Figure 22d, respectively. Compared to the cladding plates utilizing the A6005C aluminum alloy (Figure 22c,d), the thickness of the interlayer in the AZ31/A5052 and AZ80/A5052 cladding plates (Figure 22a,b) is larger, with the thickest part measuring approximately 2.0 µm and 6.0 µm, respectively. Thus, it is clear that the thickness of the interlayer formed at the interface increases with the concentration of magnesium in the aluminum alloy. The chemical composition analysis revealed the formation of the γ-Mg_17_Al_12_ phase in the interlayer of the AZ31/A5052 and AZ80/A5052 cladding plates. A previous study has reported the formation of β-Al_3_Mg_2_ and γ-Mg_17_Al_12_ phases with a two-layer structure at the interface of laser-welded magnesium alloy/aluminum alloy joints, where the heat input from laser welding is larger than that from explosive welding [44]. In our study, the β-Al_3_Mg_2_ phase was not observed at the interface of any of the cladding plates.

### 5.2. Shear Strength

**Figure 23:**
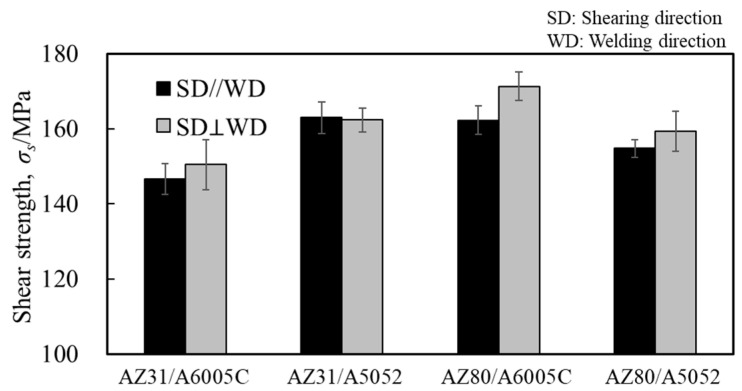
Shear strength for explosively welded AZ31/A5052 and AZ80/A5052 cladding plates.

**Figure 24:**
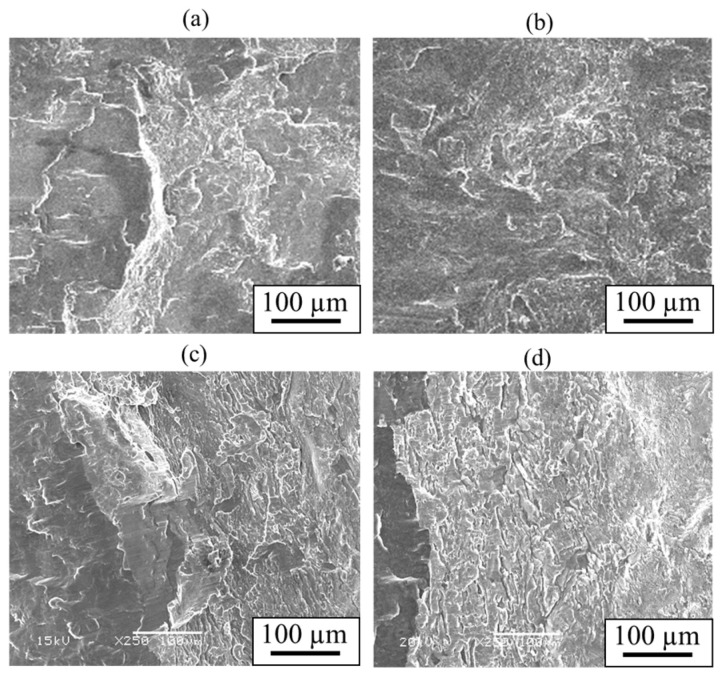
Fracture surface after shear test for explosively welded (**a**) AZ31/A5052, (**b**) AZ80/A5052, (**c**) AZ31/A6005C, and (**d**) AZ80/A6005C cladding plates.

Similarly to Section 4.3, shear tests were conducted. Figure 23 shows the obtained shear strength, including the results for the cladding plates using A6005C for comparison. The shear strength increased to the order of AZ31/A6005C, AZ31/A5052, AZ80/A6005C, and AZ80/A5052. The shear strength of the AZ31/A5052 cladding plate, which had a thick and uniform interlayer, was higher than that of the AZ31/A6005C cladding plate, which had a thin and intermittent interlayer. Conversely, the shear strength of AZ80/A5052, which had a thicker interlayer, was lower than that of AZ80/A6005C. In other words, the shear strength decreases when the thickness of the interlayer exceeds a certain value. Similarly to the results in Figure 17, the shear strength tended to be higher when the SD was parallel to the WD than when it was perpendicular to it. The fracture surface of the specimens on the magnesium alloy side of the AZ31/A5052 and AZ80/A5052 cladding plates after the shear test are shown in Figure 24a and Figure 24b, respectively. For comparison, the results of the AZ31/A6005C and AZ80/A6005C cladding plates are also shown in Figure 24c and Figure 24d, respectively. In all samples, the cross-section exhibited a cleavage fracture, indicative of a brittle fracture, and the river pattern did not display a fixed direction, indicating unclear crack propagation. Traces of the interlayer were found on the fracture surface, suggesting that the cracks may have originated there during the shear test. Regarding the shape of the wavy interface, the amplitude of the cladding plates using A5052 is smaller than that of the cladding plates using A6005C, suggesting that the shear strength is influenced by the anchor effect. Further investigation is needed to explore the relationship between the wavy interface and the shear strength.

In a study using the FSW method, it was reported that the tensile strength significantly decreased when the thickness of the interlayer was 1 μm or more [45]. In our investigation, comparing AZ31/A6005C and AZ80/A5052, in which the thickness of the interlayer is up to 6 μm, exhibits higher shear strength, although an accurate comparison is required to clarify the effect of the joining method on mechanical properties using the same test method. Regarding the shear strength of the explosively cladded plates, in addition to the thickness of the interlayer, it is presumed that the morphology of the interlayer (intermittent and non-uniform or uniform formation) and the mechanical properties of the base and flyer plates used for explosive welding also have an effect.

## 6. Effect of Annealing After Explosive Welding

In the previous sections, the interfacial microstructure after explosive welding was evaluated, although the thermal stability of this interfacial microstructure is not clear. Therefore, in this section, the effects of annealing treatment after explosive welding on the interfacial microstructure, shear strength, and nano-hardness at the interface are evaluated specifically for the AZ80/A6005C cladding plate that has already been addressed in the previous sections. Annealing was conducted at 373 K and 473 K for 0.5 h or 24 h using an air furnace. After annealing, the samples were placed on a heat-resistant material and allowed to cool. The chemical compositions of the extruded materials are shown in Table 4.

### 6.1. Characteristics of the Interlayer at the Interface

**Figure 25:**
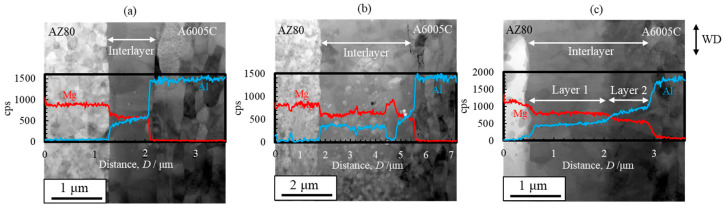
STEM images and line profiles of magnesium and aluminum compositions near the bonding interface of explosively welded AZ80/A6005C cladding plates ((**a**) as welded, (**b**) annealed at 373 K, and (**c**) annealed at 473 K for 24 h) [16]. (The figure is modified from the original version).

STEM images and line profiles of magnesium and aluminum compositions near the bonding interface of the AZ80/A6005C cladding plates with the as welded, annealed at 373 K, and annealed at 473 K conditions are shown in Figure 25a, Figure 25b, and Figure 25c, respectively. Annealing led to an increase in the thickness of the interlayer at the interface to approximately 2–4 μm. When the cladding plates were annealed at 373 K, the interlayer partially thickened, whereas at 473 K, it uniformly thickened at the interface. Additionally, while the interlayer consisted of a single layer at 373 K, it exhibited a two-layer structure at 473 K. The chemical composition analysis clarified the presence of the γ-Mg_17_Al_12_ phase in the samples both before and after annealing at 373 K, while a new Al-rich β-Al_3_Mg_2_ phase was formed on the A6005C side after annealing at 473 K.

### 6.2. Shear Strength

**Figure 26:**
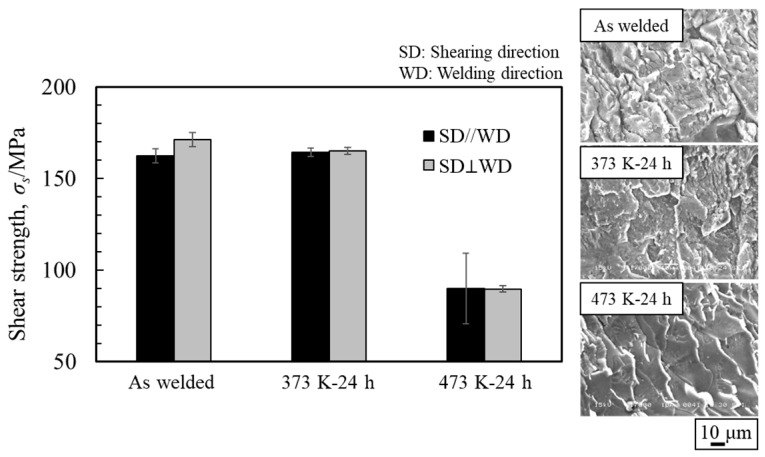
Shear strength and fracture surface after shear test for explosively welded AZ80/A6005C cladding plates with the as welded, annealed at 373 K, and annealed at 473 K conditions [16] (The figure is modified from the original version).

Th obtained shear strength and the fracture surface of the specimen on the magnesium alloy side are shown in Figure 26. The explosively welded sample and the sample annealed at 373 K exhibited similar shear strengths, with no anisotropy observed due to the shear test direction. Conversely, for the sample annealed at 473 K, the shear strength decreased significantly in all shear directions, and it was lower when the shear and bonding directions were parallel. In all samples, the cross-section exhibited a cleavage fracture, indicative of a brittle fracture, with a particularly clear crack propagation direction observed in the sample annealed at 473 K. Traces of the interlayer were identified on the fracture surface, suggesting that cracks originated from there during the shear test.

### 6.3. Nano-Hardness Across the Interface

**Figure 27:**
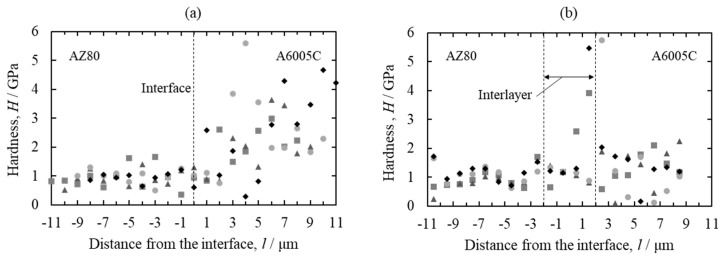
Profiles of the nano-hardness across the interface for explosively welded AZ80/A6005C cladding plates ((**a**) as welded and (**b**) annealed at 473 K for 24 h). Each symbol represents each measurement line [16].

To investigate the nanomechanical properties of the interface of explosively welded cladding plates, nanoindentation tests were conducted on the AZ80/A6005C cladding plate before and after annealing. A maximum load of 1.5 × 10^−3^ gf was applied with a load holding time of 1 s. Nanoindentation tests were performed across the interface, with measurements taken in four rows, each containing 20 points. The results are depicted in Figure 27. Before annealing (Figure 27a), the hardness exhibited greater variation on the aluminum alloy side compared to the magnesium alloy side. Following annealing at 473 K (Figure 27b), the hardness of both the magnesium alloy and the aluminum alloy decreased. In the interlayer at the interface, the hardness was nearly identical to that of the magnesium alloy side before annealing. However, after annealing, the hardness of the interlayer notably increased, particularly on the aluminum alloy side. As observed in Figure 25, the interlayer exhibited a two-layer structure after annealing at 473 K, with the formation of the β-Al_3_Mg_2_ phase on the aluminum alloy side. Su et al. reported the formation of the γ-Mg_17_Al_12_ phase and β-Al_3_Mg_2_ phase at the interface of the AZ31 magnesium alloy/A6061 aluminum alloy cladding plates obtained by ultrasonic welding [46]. Hardness measurements produced values of 3.4 GPa and 6.5 GPa for the γ-Mg_17_Al_12_ phase and β-Al_3_Mg_2_, respectively, consistent with our findings. As demonstrated in Figure 26, the shear strength decreased significantly after annealing at 473 K. Based on these results, it is inferred that the hardness of the β-Al_3_Mg_2_ phase is high and brittle, potentially serving as the site of initiation of the fracture, thus reducing the shear strength.

## 7. Future Challenges

Explosive welding allows instantaneous joining of metallic materials and achieves joining of large areas, and this study has shown that it is also effective for joining magnesium alloys and aluminum alloys. The practical application of this technology is now under consideration. Based on the research results described in the previous sections, the following are future issues to be addressed.

(1) Clarification of formation and growth mechanisms of the interlayer

It is essential to elucidate the formation and growth mechanisms of the β-Al_3_Mg_2_ and γ-Mg_17_Al_12_ phases. Additionally, further investigation is required into the dynamic recrystallization behavior during the cooling process and the associated phase growth.

(2) Detailed Analysis of Recrystallization Behavior and Grain Size Changes

Regarding the grain refinement of AZ31, detailed experiments related to recrystallization behavior and phase transformations are necessary. Further experiments are required to analyze the effects of stress and temperature on the grain structure.

(3) Shear Strength and Failure Mechanisms

While shear strength has been observed to increase with aluminum content, further experiments are necessary to understand the failure mechanisms, especially at the bonding interface. Studies focusing on crack propagation and the influence of the interlayer on fracture toughness will provide a clearer understanding of how to achieve high-strength cladding plates.

(4) Corrosion Mechanisms and Behavior

Further studies are needed to understand the corrosion mechanisms of explosively welded cladding plates, particularly the relationship between alloy composition, galvanic corrosion, and the electrochemical behavior of the interface. Understanding how varying aluminum concentrations influence corrosion potential will aid in designing more corrosion-resistant cladding plates.

(5) Detailed Evaluation of the Effects of Annealing Treatment

To deepen the understanding of how annealing treatment affects the interlayer structure and mechanical properties, evaluations under different temperature conditions and treatment times should be carried out. Finding the optimal annealing conditions is crucial.

By addressing these challenges, the explosive welding process can be optimized for a wider range of magnesium–aluminum alloy combinations, improving both the mechanical and corrosion properties of the welded joints.

## 8. Conclusions

In this review, the characteristics of explosively welded magnesium alloy/aluminum alloy cladding plates are discussed. The summary of this review is as follows:

(1) A wavy interface was observed at the bonding interface, resulting from the shear flow parallel to the direction of explosive welding and displacement restraint in the vertical direction. On the magnesium alloy side, fine recrystallized grains were generated by explosive welding.
(2) At the interface of the AZ31/A6005C cladding plate, a thin interlayer was formed, and the presence of the γ-Mg_17_Al_12_ phase was confirmed within it. The thickness of the interlayer was non-uniform, ranging from 30 to 95 nm. As the aluminum concentration in the magnesium alloy increased, the thickness of the interlayer also increased. The thicknesses in the AZ61/A6005C and AZ80/A6005C cladding plates were approximately 0.5 μm and 0.7 μm, respectively. Moreover, as the magnesium concentration in the aluminum alloy increased, the thickness of the interlayer further increased. The thicknesses in the AZ31/A5052 and AZ80/A5052 cladding plates were approximately 2.0 µm and 6.0 µm, respectively.
(3) Shear strength increased in the order of AZ31/A6005C, AZ31/A5052, AZ80/A6005C, and AZ80/A5052. The AZ80/A6005C cladding plate, where an interlayer was uniformly formed at the interface, exhibited a higher shear strength than the AZ31/A6005C cladding plate, where a thin, uneven interlayer was present at the interface. In contrast, the shear strength of AZ80/A5052, which had a thicker interlayer, was lower than that of AZ80/A6005C. Thus, the shear strength decreases when the thickness of the interlayer exceeds a certain value.
(4) Regarding corrosion resistance, the weight loss of the corrosion of the explosively welded cladding plates was slightly smaller than that of the mechanically fastened samples, indicating that corrosion progresses more easily with the mechanically fastened samples than with the explosively welded cladding plates.
(5) Annealing for the AZ80/A6005C cladding plates increased the thickness of the interlayer to about 2–4 μm. The γ-Mg_17_Al_12_ phase -Mg_17_Al_12_ was observed in the samples before annealing and after annealing at 373 K, and a new Al-rich β-Al_3_Mg_2_ phase was formed on the A6005C side after annealing at 473 K. The β-Al_3_Mg_2_ phase was hard and brittle, acting as a fracture initiation site and consequently reducing the shear strength.

## Figures and Tables

**Table 1:** Chemical compositions of the investigated alloys in Section 3 (mass %).

Sample	Mg	Al	Zn	Si	Mn	Cu	Fe	Ni
AZ31	Bal.	3.0	0.9	0.02	0.3	0.002	0.004	<0.002
A6005C	0.6	Bal.	0.00	0.6	0.01	-	0.1	-

**Table 2:** Chemical compositions of the investigated alloys in Section 4 (mass %).

Sample	Mg	Al	Zn	Si	Mn	Cu	Fe	Ni
AZ31	Bal.	3.0	0.9	0.02	0.3	0.002	0.004	<0.002
AZ61	Bal.	5.7	0.7	0.02	0.3	<0.002	<0.002	<0.002
AZ80	Bal.	8.0	0.6	0.03	0.3	<0.002	0.002	<0.002
A6005C	0.6	Bal.	0.00	0.6	0.01	-	0.1	-

**Table 3:** Chemical compositions of the investigated alloys (mass %).

Sample	Mg	Al	Zn	Si	Mn	Cu	Fe	Ni
AZ31	Bal.	3.0	0.9	0.02	0.3	0.002	0.004	<0.002
AZ80	Bal.	8.0	0.6	0.03	0.3	<0.002	0.002	<0.002
A6005C	0.6	Bal.	0.00	0.6	0.01	-	0.1	-
A5052	2.58	Bal.	0.02	0.08	0.02	-	0.27	-

**Table 4:** Chemical compositions of the investigated alloys (mass %).

Sample	Mg	Al	Zn	Si	Mn	Cu	Fe	Ni
AZ80	Bal.	8.0	0.6	0.03	0.3	<0.002	0.002	<0.002
A6005C	0.6	Bal.	0.00	0.6	0.01	-	0.1	-
